# Energy budget and carbon footprint assessment under diverse nitrogen management modules in mustard (*Brassica juncea* L.) production under subtropical climate

**DOI:** 10.1371/journal.pone.0332754

**Published:** 2025-10-23

**Authors:** Vasudev Meena, Mohan Lal Dotaniya, Murli Dhar Meena, Ram Swaroop Jat, Mukesh Kumar Meena, Ram Lal Choudhary, Hari Singh Meena, Bheeru Lal Meena, Prabhu Dayal Meena, Pramod Kumar Rai, Vijay Veer Singh

**Affiliations:** 1 ICAR-Indian Institute of Rapeseed-Mustard Research, Bharatpur, India; 2 ICAR - National Institute of Biotic Stress Management, Raipur, India; University of Delhi, INDIA

## Abstract

In the context of a changing climate, identifying a sustainable food production system that incorporates cleaner technologies with low C-sequestration and minimal energy inputs is crucial for long-term sustainability. The objective of study was to develop an innovative, energy-efficient system for mustard cultivation with reduced carbon footprint and economic viability by optimizing nitrogen (N) management. The experiment included nine nitrogen management strategies plus one unfertilized treatment as control, arranged in a randomized complete block design in three replicates. Results demonstrated that sensor-based nitrogen application using the GreenSeeker (GS) significantly augmented economic yield by 19.3% and 64.5%, and proved more profitable, boosting net monetary returns and benefit-cost ratio by 125.1% & 36.2% and 58.8% & 24.4%, respectively compared to the recommended dose of fertilizer (RDF) and control, and saved 18.7% of nitrogen. The yield of mustard seeds increased significantly, ranging from a minimum of 3.70% (with RDN_75_ + foliar spray @ 1.5% KNO₃) to a maximum of 19.31% under the GreenSeeker (GS) treatment. Further, N foliar spray treatments at N_100_ level registered for per cent negative changes in N efficiency (−6.90 to −1.46%) over RDF. Nearly half (46.25%) of the total energy consumption was attributed to fertilizer nitrogen, diesel fuel, threshing, and irrigation contributing 17.7%, 11.4%, 9.68%, and 7.40%, respectively. The GS guided N application consumed comparably lowest energy (5.91% less) than RDF. The energy indices *viz.* energy input (−5.98%), energy output (+7.25%), energy use efficiency (6.51%), energy profitability (5.51), energy productivity (+18%), respectively were achieved higher by precise N administration using sensor based GS. In contrast, the specific energy (1.39 MJ kg^-1^), energy intensiveness, direct and non-renewable energy usage were highest under RDN_100_ over RDN_75_ in conjunction with FS of different N sources (U, NCU and KNO_3_). Congruently, human energy profitability was varied from 0.34 to 9.78%, respectively over RDF. Carbon-related metrics showed that RDN_100_ produced higher carbon inputs, outputs, net carbon gains, and spatial carbon footprints compared to RDN_75_. However, GS-based management outperformed RDF, with lower carbon input (−8.1%), higher carbon output (+10.9%), greater net carbon gain (+16.5%), and the lowest carbon footprint (0.30 kg CE kg ⁻ ¹) versus RDF 0.39 kg CE kg ⁻ ¹. Furthermore, CO₂ emissions were approximately 81% higher in fertilized plots (1921 kg CO₂-e ha ⁻ ¹) compared to unfertilized ones. Overall, the study concludes that sensor-based precise nitrogen management using GS is an innovative, sustainable, and energy-efficient approach that reduces the carbon footprint, combat climate change, and supports food security.

## Introduction

The increasing fluctuations in climate, along with reduced soil fertility, shrinking land holdings, and poor productivity, have heightened the global worries about feeding the growing population [1,2]. The agriculture sector as a second-biggest source of greenhouse gas emissions (GHGs) accounting for 17.6% of all [3,4]. Among these GHGs, CO_2_, CH_4_ and N_2_O accounted for 13, 60 and 50% emission, respectively [5]. The agricultural inputs namely manures and fertilizers as nutrients sources, fuel, irrigation, machineries, herbicides, and pesticides are considered as main sources of GHGs emissions [6]. Overexploitation of resources coupled with reckless nutrient usage and declining soil fertility, soil erosion, acidity and salinity cause reduction in the Nutrient Use Efficiency (NUE), thereby enhancing overall plant nutrients necessity [7]. As we all know that the nutrients, energy & water are the primary requirement in any of the food producing system and their appropriate management results into highest recovery. Further in nutrients, nitrogen is one of the most dynamic both in soil as well as plant system, as evidenced by their low N recovery (<50%) in a variety of crops [8–10]. Applying fertilizer N alone persistently results in a decrease in soil pH and nutrient delivery capability gradually and it is linked to soil compaction and structural degradation [11]. Additionally, the overuse of chemical fertilizers negatively impacts crop yields, degrades soil health, and harms the environment, as it is a significant source of N₂O emissions [12,13] in addition to being an expensive agricultural input to produce and use both economically and environmentally [14]. The microorganisms that are engaged in the nitrification and denitrification processes produce N_2_O, an active greenhouse gas that significantly increases global greenhouse gas emissions and the carbon footprint [15]. According to Reay et al. [16] approximately 80% of the N_2_O emission generated from agricultural sector caused by the nutrients sources applied to the soil during crop cultivation and it is getting worse with more nitrogen being supplied, endangering the sustainability of the soil and environment [17]. Therefore, the biggest obstacles to preserving higher production potential, economic feasibility, environmental sustainability, and societal well-being are poor nutrient management techniques [18]. Additionally, worldwide land use pattern is equally responsible (10–12%) because of anthropogenic yearly greenhouse gas emissions [19].

Fossil fuel combustion is the primary source of energy in farming practices which accounts for a significant portion of greenhouse gas emissions [20,21]. Fossil fuels are vital to the production of food since they are used as fertilizers, agrochemicals derived from petroleum, and fuel for farm machinery upkeep [22]. Since fertilizer production and transportation account for the biggest share of all energy inputs, they are recognized as significant contributors to greenhouse gas emissions [23]. In addition, the energy consumed for a variety of agricultural activities contributes to GHGs release that has a negative impact on the environment [24]. An alternate strategy that could lessen the negative stuffs of synthetic nutrient sources besides maintaining soil fertility and enhancing ecological and agronomic benefits is through adopting appropriate management of organic carbon [25].

Owing to growing oil consumption and diversion as an alternate source of bioenergy, the demand for rapeseed-mustard oil is still rising dramatically. In comparison to cereals, inappropriate nutrient management in rapeseed-mustard production is the main cause for lesser yield, lower economic viability as against their full yield potential. *Brassica* being an extremely N responsive crop require large quantity of nitrogenous fertilizer to achieve optimal yields [26]. Additionally, *Brassica* species’ inability to efficiently use nitrogen is conspicuously hampered by their high absorption and low translocation rates towards reproductive parts [27] resulting in reduced N utilization efficiency. Nitrogen splitting for realizing greater NUE is quite labour intensive and require more energies thereby decreases farm profitability as rapeseed-mustard require more N than P and K since most of the Indian soils are lacking these mineral elements. The most important prerequisites for enhancing *Brassica* oil seed production and productivity are genetic improvement in yield potential with sufficient, well-balanced nutrition. Similarly, energy is another most important agriculture input after nutrients in order to meet the expanding population’s demand for food. Being a high-energy crop, mustard is normally cultivated in a low-energy environment. More emphasis needs to be placed on the critical interaction between energies and farming systems in the current intensive cereal-based cropping systems, particularly in situations where resources are scarce.

The United Nations General Assembly adopted the Sustainable Development Goals in 2015, and it is imperative that we prioritize eradicating hunger, reducing poverty, and ensuring that everyone lives in dignity on a cleaner, safer world [28]. Consequently, in order to achieve food and nutritional security by 2030, the nation must strengthen its current food production systems in a sustainable way. This will call for a transformed and aggressive push for agricultural inputs as well as a thorough examination of a production system’s energetics, C-footprint, and cost-effectiveness in order to get a broad understanding of its environmental influence. The ability of a production systemto use energy efficiently and productively depends on the careful application of a variety of inputs, insecticides, weedicides, machineries, nutrients and electrical components. There is a positive correlation between the intensity of production inputs and the energy utilization in food production [29]. Yet, the short-term advantages of increased crop yield brought about by augmented use of non-renewable energy sources must be balanced with the long-term costs incurred by society as a result of resources’ depletion [30]. Energy balancing studies play a crucial role in developing safer and more environmental friendly clean production technologies by diminishing greenhouse gas emissions [31]. Majority of the earlier research on managing nutrients in mustard cultivation has been solely focused on few aspects like productivity, nutrient acquisition and profitability. In the existing context of global warming, mechanization, and resource-intensive farming, research studies should go beyond yield and cost analysis [32,33]. Above all, the studies need to exhibit carbon footprints and energy matrices for sustainability of production system. A carbon footprint in agriculture refers to the total amount of greenhouse gas emissions, expressed as carbon dioxide equivalents, resulting from all activities related to food production, from farm to fork. Reducing agriculture’s carbon footprint is crucial for mitigating climate change impacts and ensuring food security for a growing global population. It is crucial to develop an alleviation tactic framework that co-optimizes lesser energy consumption and emission cut-off in order to minimize the global warming potential besides enhancing environmental sustainability. A technology with cleaner crop production and energy efficient in mustard, having minimal C-footprint is required and until now, there has not been much research on mustard nutrient management in relation to energy budgeting and C-footprints. Adopting low-carbon practices can lead to cost savings for farmers, such as reducing energy consumption and improving fertilizer efficiency. Additionally, the development and adoption of sustainable technologies and practices can create new economic opportunities in the agricultural sector.

Henceforth, the objective behind the study was finding out of an innovative and sustainable crop N management tactic under climate change era concerning C-sequestration with reduced energy consumption encompassing cleaner production. In this way, an attempt has been made for assessing energy and C-footprints of diverse N management options to determine their efficiencies in mustard production with the hypothesis that the optical sensor based optimized use of nitrogen will reduce the carbon footprint and mitigate the global climate change impacts with sustainable mustard production.

## Materials and methods

### Site description and soil type

A field study was conducted at research farm of ICAR-Directorate of Rapeseed-Mustard Research, located at Bharatpur with latitude and longitude of 27°12’8.9“ N and 77°27’18.8” E at 178.4 m above MSL altitude) during winter season for two consecutive years using *Brassica juncea* L. as test crop. The region has a subtropical, semi-arid climate, receiving approximately 650 mm of annual rainfall, with 85–90% occurring between July and September due to the southwest monsoon, and occasional light winter showers in January. Before sowing, soil samples were taken, and their physico-chemical characteristics were examined (Table 1). The soil’s texture was described as clay loam, and it had low levels of accessible nitrogen (126.3 kg ha^-1^) and organic carbon (2.4 g kg^-1^). It also had medium levels of extractable phosphorus (17.2 kg ha^-1^) and exchangeable potassium (1.0 N NH4OAc, 149.3 kg ha^-1^). In reaction, the experimental field’s soil pH was alkaline (8.3) with an EC of 1.30 dS m^-1^.

**Table 1:** Physico-chemical properties of experiment soils.

Soil characteristics	Value
*Physical properties*	
Coarse sand (%)	21.5
Fine sand (%)	54.2
Silt (%)	16.2
Clay (%)	8.2
Textural class	Loamy sand
EC (dSm^-1^)	1.3
pH (1:2 soil water suspension)	8.3
Bulk density (mg m^-3^)	1.52
Particle density (mg m^-3^)	2.5
Field capacity (%)	12.5
Permanent wilting point (%)	2.4
*Chemical properties*	
Organic carbon (g kg^-1^)	2.4
Avalaible N (kg ha^-1^)	126.3
Avalaible P (kg ha^-1^)	17.2
Avalaible K (kg ha^-1^)	149.3
SO_4_^−2^ (mg kg^-1^)	8.3

### Experimental setup and crop husbandry

The experimental unit was prepared by giving irrigation prior to seeding. The randomized complete block design was used for the experiment, and there were three replications incorporating ten treatments. The mustard variety *DRMR 2015–17* was sown with the help of seed drill in the last week of October in both the years (29.10.2021 and 21.10.2022). The seeds were placed in the furrow at a depth of 2–3 cm with 45 cm row to row and 15 cm plant to plant distance following the seed rate of 3.5 kg ha^-1^. To maintain the ideal plant population gap filling was carried out at 4–6 DAS. A uniform application of recommended dose of fertilizer *viz*. N, P_2_O_5_, K_2_O, S and B were applied at the rate of 80-40-40-40-1 kg ha^-1^, respectively to the crop by using urea, SSP, MOP, sulphur and borax. To ensure uniformity across treatments, half of the total nitrogen and the full recommended dose of PKSB were applied as a basal dose to each plot at the time of sowing. The remaining half of the nitrogen (50%) was top dressed at the time of first irrigation, i.e., 30–35 days after sowing in accordance with specific treatments using different N management techniques designed to satisfy the crop’s unique nutritional requirement. In order to maintain spacing of 10 cm between the plants in a row, excess plants were removed during thinning operation, which took place between 18 and 21 DAS. Aside from managing nutrients, all inter-culture techniques were maintained constant throughout the crop-growing period in every plot. Once the crop reached at physiological maturity, it was reaped manually in the month of March and April. The crop was then dried and threshed, and the seed output from each experimental unit was recorded independently.

### Study of parameters

Data on diverse growth and yield characteristics *viz*. plant tallness, per plant number of branches, siliquae number and their length, number of seed in each siliquae, thousand seed weight, economic and biomass yield were documented using standard procedures at harvest.

### Energy calculation

A comprehensive inventory of used agricultural inputs and outputs during crop cultivation was prepared to determine the energetics of various nitrogen management modules, including land preparation, seeds and sowing, fertilizers, weed management, diesel, man power, machineries, seed output and biomass [31,34–40]. Energy coefficients from published literature were utilized to convert the physical units of all used agricultural inputs and outputs into energy units (Table 2). To arrive overall energy input, the energy input for each process and the inputs employed in crop cultivation were added together. In a same vein, the evaluation of energy output took into account the mustard seed plus stover yield. The below mentioned formulas were used to determine the different energy parameters in accordance with the methods recommended by Mittal and Dhawan [41].

**Table 2:** Energy equivalent of agricultural operations utilized during the experimentation as both inputs and outputs.

Inputs	Unit	Energy coefficient	Reference
Tractor 50 HP	hr	64.8	Parihar et al. (2018)
Plough	hr	62.7	Parihar et al. (2018)
Cultivator	hr	22.8	Dagistan et al. (2009)
Rotavator	hr	6.69	Kitani (1999)
Furrow opener	hr	22.8	Dagistan et al. (2009)
Sprayer	hr	0.17	Kitani (1999)
Human labour	hr	1.96	Parihar et al. (2018)
N	kg	60.6	Parihar et al. (2018)
P_2_O_5_	kg	11.1	Parihar et al. (2018)
K_2_O	kg	6.7	Parihar et al. (2018)
S	kg	1.12	Yadav et al. (2017)
Zinc sulphate	kg	20.9	Chaudhary et al. (2017)
Seed	kg	20.4	Campose et al. (1998)
PP chemicals	l	150.9	Green (1987)
Insecticides	kg	184.63	Parihar et al. (2018)
Diesel	l	56.31	Parihar et al. (2018)
Irrigation	m^3^	1.02	Mohammadi et al. (2008)
Water for spray	m^3^	0.17	Mandal et al. (2002)
Harvesting labour	hr	1.57	Parihar et al. (2018)
Thresher	hr	68.4	Nassiri and Singh (2009)
**Output**			
Seed	kg	22.72	Yadav et al. (2017)
Stover	kg	12.5	Yadav et al. (2017)


$$EC=\sum_{i=\text{1}}^{n}\left(E\text{1}+E\text{2}+\ldots ..En\right)$$

EO=Y X E

$$EUE\;(\%)\;=\frac{EO}{EC}$$

$$EP\;(kg\;MJ¯¹)=\sum_{i=\text{1}}^{n}\left(\frac{Y}{EC}\right)$$

$$SE\;(MJ\;kg¯¹)=\sum_{i=\text{1}}^{n}\left(\frac{EC}{Y}\right)$$

$$NE\;(MJ\;ha{¯}^{\text{1}})=EO-EC$$

$$EI\;(MJ\;USD{¯}^{\text{1}})=\frac{EC}{CC}$$

$$HEP=\frac{EO}{Labour\;energy}$$

$$EPfy=\frac{NE}{EC}$$

 DE=Labour+Fuel

IDE=Seeds+ Fertilizers+Chemicals+Machineries

RE=Labour+Organic manure

NRE=Fuel+Electicity+Seeds+ Fertilizers+Chemicals+Machineries

Here, EC: energy consumption in MJ ha^-1^; E1 + E2…..En, indicating energy of specific components (MJ ha^-1^); EO: energy output; EUE: energy use efficiency; EP: energy profitability; SE: specific energy; NE: net energy; EI: energy intensive; HEP: human energy profitability; DE: direct energy; IDE: indirect energy; RE: Renewable energy; NRE: non-renewable energy; Y: biological yield (t ha^-1^); CC: cost of cultivation (USD ha^-1^). Primary data related to inputs and agronomic practices were used to calculate the overall energy consumption. The above indices are relevant for assessing changes in energy requirements influenced by different treatments.

### Carbon footprint

The carbon footprint was computed to distinguish the greenhouse gas emission potential of various nitrogen management modules used in the current investigation. The greenhouse gases (GHGs) generated over course of the growing season, measured in CO_2_ equivalents (CO_2_-e), make up the total carbon footprint [42]. The carbon emission factor determined by [43–46] as shown in Table 3, was used to estimate carbon dioxide, weedicide, insecticide, and fungicide. The estimates were expressed in units of kg CO_2_-eq ha^-1^. By adding together the CO_2_ emissions from various inputs, the total CO_2_ emission was calculated. Special carbon footprint is the equivalent, expressed in kilograms CO_2_-e ha^-1^, on an area basis. According to Kumar et al. [47] recommended approach, the total amount of CO_2_ released under diverse treatments were split by dividing 3.66 to derive equivalent carbon (kg CE ha^-1^) from a special carbon footprint (kg CO_2_-e ha^-1^). The total carbon output in mustard biomass was estimated by multiplying 0.44 (assuming that the biomass contains 40% carbon) with the economic yield [31]. Utilizing the methods proposed by Lal [45], various C-indices were calculated including C-output, C-gain, carbon efficiency, and carbon sustainability index.

**Table 3:** Emission factors (carbon equivalent) of agriculture inputs used in the estimation in the study.

Inputs	Units	Equivalent energy (MJ)	References
Plough	hr	31.97	West and Marland (2002)
Cultivator	hr	4.00	Lal (2004)
Rotavator	hr	2.00	Lal (2004)
Furrow opener	hr	24.90	West and Marland (2002)
Human labour	days	0.86	Deng (1982)
N	kg	4.96	Lal (2004)
P	kg	1.35	Lal (2004)
K	kg	0.58	Lal (2004)
S	kg	0.30	Mohammadi et al. (2008)
Herbicide/Pendi	liter	6.30	Lal (2004)
Insecicide-Profenofos	liter	5.10	Lal (2004)
Propiconazole	liter	3.90	Lal (2004)
Machinary	hr	3.32	Deng (1982)
Electricity	KwH	0.608	Khodi and Monsavi (2009)
Diesel	liter	3.32	Deng (1982)
Irrigation	m^3^	1.03	Zahedi et al. (2015)
Seed	kg	1.22	Wang et al. (2015)


$$Carbon\;footpriniting\;=\frac{Carbon\;emmission\;\left(kg\;CE\;ha{¯}^{\text{1}}\right)}{Mustard\;seed\;yield\;\left(kg\;ha{¯}^{\text{1}}\right)}$$

$$Carbon\;output\;\left(kg\;CE\;ha{¯}^{\text{1}}\right)=Total\;biomass\;(Economic\;yield+biproduct\;yield)\;\times \;\text{0}.\text{4}\text{4}$$

$$Net\;carbon\;gain=Carbon\;output\;\left(kg\;CE\;ha{¯}^{\text{1}}\right)-\;Carbon\;input\;\left(kg\;CE\;ha{¯}^{\text{1}}\right)$$

$$Carbon\;efficiency\;ratio\;=\frac{Carbon\;output\;\left(kg\;CE\;ha{¯}^{\text{1}}\right)}{Carbon\;input\;\left(kg\;CE\;ha{¯}^{\text{1}}\right)}$$

$$Carbon\;efficiency\;\left(kg\;kg{¯}^{\text{1}}\;CE\right)=\frac{Mustard\;seed\;yield\;\left(kg\;ha{¯}^{\text{1}}\right)}{Carbon\;input\;\left(kg\;CE\;ha{¯}^{\text{1}}\right)}$$

$$Carbon\;sustainability\;index\;=\frac{Net\;carbon\;gain\;\left(kg\;CE\;ha{¯}^{\text{1}}\right)}{Carbon\;input\;\left(kg\;CE\;ha{¯}^{\text{1}}\right)}$$

$$N_{\text{2}}O\;emmission\;\left(kg\;N_{\text{2}}O\;year{¯}^{\text{1}}\right)=Total\;added\;N\;\left(kg\;ha{¯}^{\text{1}}\right)\;\times \;EF\;\times \;\frac{\text{4}\text{4}}{\text{2}\text{8}}$$

Where, the ratio of atomic weights of N_2_O to N_2_ (44/28 = 1.571) determines the conversion of N to N_2_O, or the quantity of N_2_O released per unit of N use. The equation given by Tubiello et al. [48] represents the kg N_2_O–N emitted per kg N input obtained by multiplying the emission factor (0.01) for N_2_O emissions with the amount of N applied from fertilizer.

The influence of N management options was determined by computing carbon footprint on an ecology-based spatial and yield-scale basis. The amount of emanated CO_2_ and N_2_O during the crop cycle expressed as CO_2_ equivalents (CO_2_-e) is referred as ecology based spatial carbon footprint [42]. Under anaerobic soils, where microorganisms do not have access to oxygen to receive electrons, methane is generated and in these circumstances, CO_2_ accepts electrons and transforms into CH_4_. The prerequisite for methane production is that the soil must have redox potential below −0.2V, but in our study soil has higher value of redox potential during the entire cropping cycle, i.e., more aerobic which was not favourable for CH_4_ emanations [49], and hence merely CO_2_ and N_2_O were considered. No on-farm burning of dry agri-biomass was observed in the experimental field. The CO_2_ and N_2_O released from the experimental unit were converted into a common unit of CO_2_e on multiplication of GWP potential of 1 & 298, respectively [50]. This covered every element that contributed to the production and consumption of any input and resulted in greenhouse gas emissions.

The GWP estimate made with equation:


$$GWP=(emitted\;N_{\text{2}})\;\times \;\text{2}\text{9}\text{8}+emitted\;CO_{\text{2}}$$

The CFs was worked out by using equation as given by Pandey and Agrawal [51].


$$CFs\;=\sum_{i=\text{1}}^{n}GWP\;(kg\;CO_{\text{2}}-e/ha)$$

Here, CFs refers to carbon-footprint on unit area basis


$$CFy\;=\frac{CFs}{Biological\;yield}\;(kg\;CO_{\text{2}}-e/t)$$

Where, CFy denotes per unit yield of CFs (yield-scaled carbon footprint), mustard biological yield is in t ha^-1^.

### Monetary analysis

Every treatment’s economic feasibility was determined using the current market value of the input items that were employed, such as fertilizer, seeds, and other supplies in the production. The overall cost of production included fuel for machines, labor costs for irrigation, seed, nutrients, and harvesting. The following equations were used to calculate the benefit-cost ratio (BCR) [52].


Cost of cultivation (CC) (US ha¯¹)=Input cost (USha¯¹)+Labour cost (US$ ha¯¹)

Gross monetary return (GMR) (US$ ha¯¹)=Economic yield (kg ha¯¹)  x Market price (per kg)

Net monetary return (NMR) (USha¯¹)=Gross return (USha¯¹)−CC (US$ ha¯¹)

BCR (USha¯¹)=GMR (USha¯¹)/CC (US$ ha¯¹) 

### Statistical analysis

The SAS 9.3 statistical software (IBM ver.23) was used to statistically analyze the data in order to determine treatments significance. An analysis of variance (ANOVA) was performed to establish the significance of the treatments after data on various parameters from two sequential years were averaged to get the mean. The treatment means effect were distinguished using DMRT [53] at *p = *0.05 level of significance.

## Results

### Yield attributes and yield

Significant improvement were noticed in data pertained to yield characteristics *viz*. per plant siliquae number, seed per pod, 1000 seed weight, and yields (economic & biomass) of mustard (Table 4). Sensor guided N management using GreenSeeker (GS) amplified number of siliquae per plant, seed per pod and 1000 seed weight to maximum by 39.7, 29.4 and 25.7% over unfertilized plot subsequently N application made through the leaf colour chart assessment (31.0, 25.7 and 25.6% increment). Across foliar spray treatments applied through various N sources, NCU @ 2% was outperformed over rest of the two other FS treatments (Urea @ 2% and KNO_3_ @ 1.5%) at RDN_100_ in comparison to RDN_75_. The per cent increment in economic yield was obtained 22.0 and 64.5% greater by the application of N through GS in different splits over recommended practices along with control (Fig 4c). Moreover, in case of LCC the per cent enhancement in yield was registered to 11.8% over recommended practice and 54.4% more as compared to control. Similarly, higher biomass yield was obtained in RDN_100_ + FS-NCU @ 2% followed by RDN_100_ + FS-Urea @ 2% over GS sensor guided nitrogen management.

**Table 4:** Yields attributes and yield of *Brassica juncea* L. as influenced by different nitrogen management modules (Pooled data of two years).

Treatments	N application				% N saving over RDF	Siliquae per plant (no.)	Seed per siliquae (no.)	1000 seed weight (g)	Seed yield (kg ha^-1^)	Stover yield (kg ha^-1^)	% Change in SY over RDF
	Basal	Top dressing	FS	Total N							
RDN_100_ (50% N basal + 50% N TD) + 2% Urea FS	40	40	5.52	85.52	−6.90	456^bc^	16.9^b^	5.72^a^	2637^bc^	7619^a^	12.55
RDN_100_ (50% N basal + 50% N TD) + 2% NCU FS	40	40	5.52	85.52	−6.90	465^b^	17.2^b^	5.76^a^	2729^b^	7647^a^	16.47
RDN_100_ (50% N basal + 50% N TD) + 1.5% KNO_3_ FS	40	40	1.17	81.17	−1.46	445^c^	16.8^b^	5.48^bc^	2558 cd	7358^b^	9.18
RDN_75_ (50% N basal + 25% N TD) + 2% Urea FS	40	20	5.52	65.52	18.10	432^d^	16.6^bc^	5.28^c^	2483^d^	7316^b^	5.98
RDN_75_ (50% N basal + 25% N TD) + 2% NCU FS	40	20	5.52	65.52	18.10	452^bc^	16.8^b^	5.59^b^	2547 cd	7169^bc^	8.71
RDN_75_ (50% N basal + 25% N TD) + 1.5% KNO_3_ FS	40	20	1.17	61.17	23.54	422^d^	16.4^c^	5.19 cd	2407^d^	6885^c^	2.73
RDN – Green seeker	40	25	–	65	18.75	517^a^	17.6^a^	5.81^a^	2859^a^	7447^a^	22.02
RDN – LCC	40	32	–	72	10.00	485^b^	17.1^b^	5.71^a^	2619^bc^	6966^c^	11.78
RDF	40	40	–	80	0.00	442 cd	16.6^bc^	5.13 cd	2343^d^	6943^c^	0.00
Control	–	–	–	–	–	370^e^	13.6^d^	4.62^d^	1696^e^	5328^d^	−27.61

# Means were compared using Duncan’s multiple range test (DMRT) at p < 0.05 level by mentioning different letters (a, b, c, d and e) in each column.

Abbreviations: RDN: recommended dose of nitrogen, TD: top dressing; FS: foliar spray, NCU: neem coated urea, RDF: recommended dose of fertilizer, LCC: leaf colour chart, SY: seed yield

**Fig 1:**
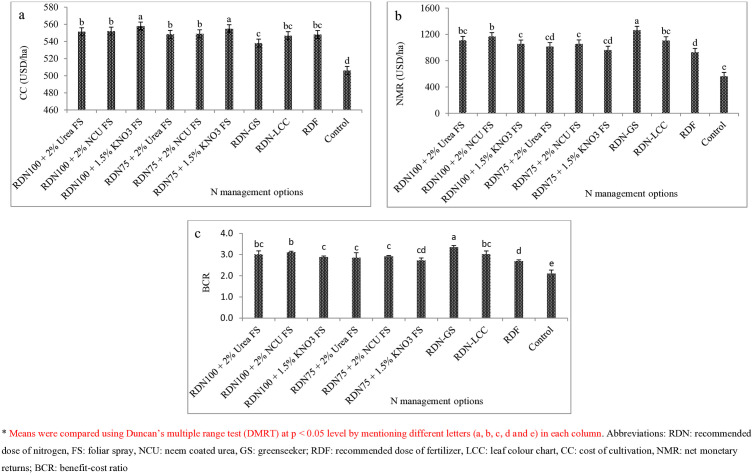
Effect of N management options on profitability of different treatments in mustard a) cost of cultivation (CC); b) NMR; c) BCR.

**Fig 2:**
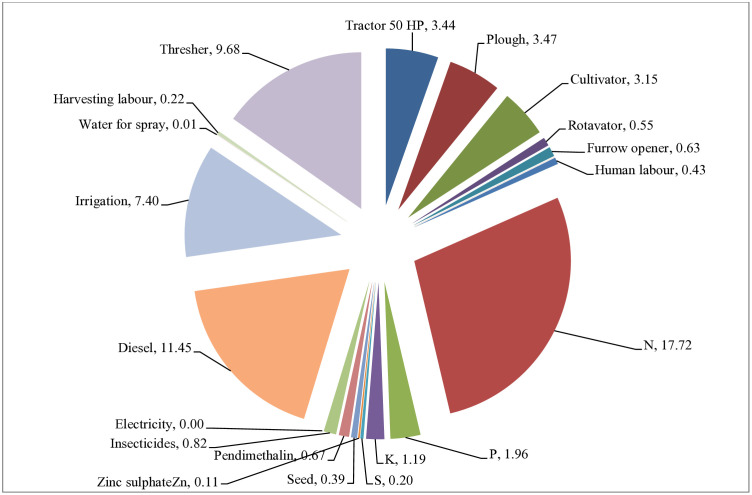
Mean energy consumption pattern across the different nitrogen management modules.

**Fig 3:**
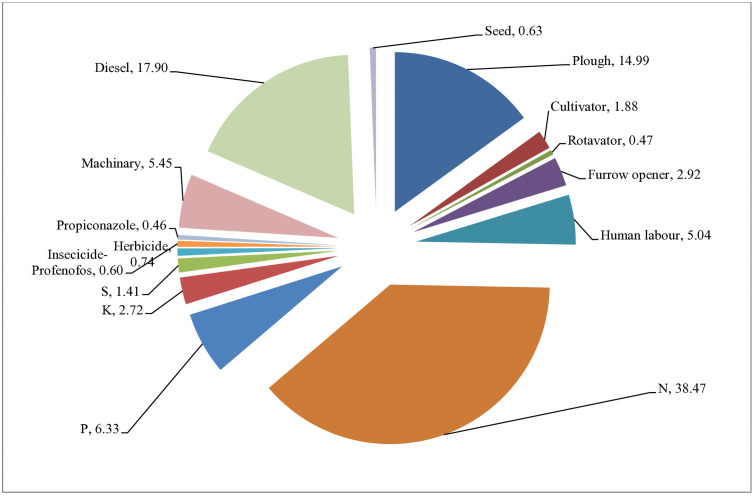
Treatment wise percent contribution of different agricultural inputs in total CO_2_ emissions.

**Fig 4:**
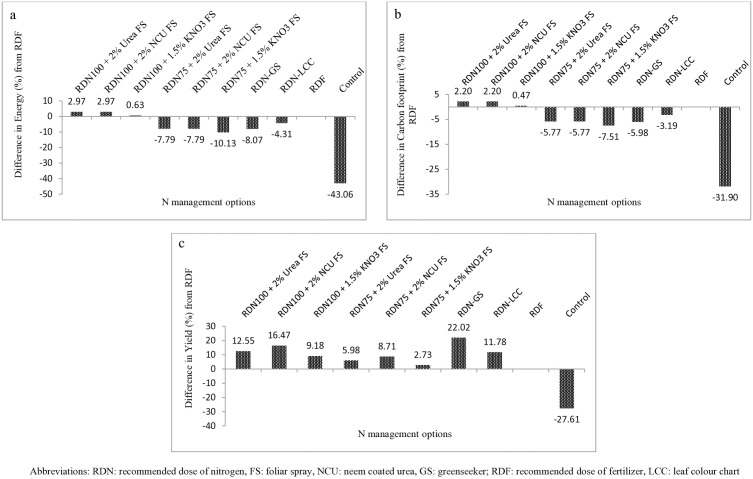
Energy saving (a); carbon footprint reduction (b) and yield variation (c) over recommended practice (RDF) under various N management options in mustard. *(+)ve values indicates increase (more requirement) and (-)ve values shows reduction (less requirement).

Overall, yield characteristics and yields at RDN_100_ were higher for all N management options compared to RDN_75_. In addition, the per cent N saving was greater (18.7%) in treatment in which N was given using Green Seeker because of appropriate N administration as compared to recommended dose (80 kg N/ha) (Table 4). Treatments with foliar application of N plus N_100_ (first three in table) registered for negative per cent changes in N (−1.46 to −6.90%) in comparison to recommended practice because of applying excess N in the form of foliar spray. However, highest per cent N saving was obtained in RDN_75 _+ FS @ 1.5% KNO_3_ due to reduced nitrogen levels as 75% of RDF plus FS (61.17 kg N/ha) as compared to recommended dose. The percent enhancement in economic yield of *Brassica juncea* L. ranged from 3.70% (lowest improvement, achieved with RDN_75_ + FS at 1.5% KNO3) to 19.31% (highest improvement, achieved with GS treatment). The unfertilized plots, on the other hand, showed a significant decline in seed yield, with a decrease of 27.46% compared to the treated plots.

### Monetary analysis

Application of diverse N management modules exhibited significant influence on treatment’s economic feasibility and their viability (*viz*. cost of cultivation-CC, net monetary return-NMR, benefit-cost ratio-BCR). The lowest expenditure in respect of mustard production was observed under Green Seeker guided multi-split nitrogen application (US$ 538/ha) over the various treatments excluding control where no fertilizers were applied (Fig 1). All the FS treatments have increased production cost as compared to GS nitrogen application. Among foliar sprays, N applied through KNO_3_ at both N levels (RDN_100_ & RDN_75_) accomplished greater cost of production. Conversely, N administration by using Green Seeker crop sensor realized higher values of NMR and BCR followed by RDN_100_ plus foliar spray of nitrogen through neem coated urea @ 2% and leaf colour chart. In GS guided N application, the percentage increase in NMR and BCR was 125.1 & 36.2% and 58.8 & 24.4%, respectively, compared to the unfertilized treatment and recommended practice. After GS, FS treatments achieved better monetary returns in terms of NMR and BCR over RDF, whereas control had lesser values of NMR and BCR (US$ 561/ha & 2.11).

### Energy consumption pattern

The energy utilization trends of *Brassica juncea* L. was significantly influenced because of application of different nitrogen management tactic (Table 5). Among the N application methods, use of fertilizer nitrogen, diesel, thresher and irrigation exhibited greatest energy utilization in mustard cultivation. Almost half of the total energy utilization (46.25%) was contributed by these four above mentioned inputs. Considering the overall energy input on average, approximately 17.7% was accounted by nitrogen, 11.4% through diesel, 9.68% by thresher, and 7.40% by irrigation (**Fig 2**). While concerning on treatment wise energy input contribution, the treatments with higher quantity of nitrogen fertilizer revealed maximum consumption. Notably the treatment combination of RDN_100_ with FS of urea or NCU @ 2% was surpassed RDN_75_ + FS of urea or NCU @ 2% and rest of the treatments in energy input consumption accounting to 15701 MJ ha^-1^ (Table 5). GS based N application consumed comparably lowest energy than RDF (5.91% less) and LCC (2.85% less) based N management module. The surplus energy consumption due to increased nitrogen levels was nearly 2.17% over the standard RDF treatment. Whereas, control treatment has consumed lowest energy (9737 MJ ha^-1^) among all the treatments because of absence of nutrient application.

**Table 5:** Energy consumption (MJ ha^-1^) for raising *Brassica juncea* L. under various nitrogen management modules (Pooled over two years).

Treatment	Energy consumption due to common management practices								Energy consumption due to treatments	Total
	Field preparation	Seed & Sowing	Weeding & thinning	Plant protection	Harvesting	Threshing	Diesel	Irrigation	Fertilizers	
RDN_100_ (50% N basal + 50% N TD) + 2% Urea FS	2542	231.75	125.44	335.53	50.24	2189	2590	1673	5965	15701
RDN_100_ (50% N basal + 50% N TD) + 2% NCU FS	2542	231.75	125.44	335.53	50.24	2189	2590	1673	5965	15701
RDN_100_ (50% N basal + 50% N TD) + 1.5% KNO_3_ FS	2542	231.75	125.44	335.53	50.24	2189	2590	1673	5701	15438
RDN_75_ (50% N basal + 25% N TD) + 2% Urea FS	2542	231.75	125.44	335.53	50.24	2189	2590	1673	4753	14489
RDN_75_ (50% N basal + 25% N TD) + 2% NCU FS	2542	231.75	125.44	335.53	50.24	2189	2590	1673	4753	14489
RDN_75_ (50% N basal + 25% N TD) + 1.5% KNO_3_ FS	2542	231.75	125.44	335.53	50.24	2189	2590	1673	4489	14226
RDN – Green seeker	2542	231.75	125.44	335.53	50.24	2189	2590	1673	4721	14458
RDN – LCC	2542	231.75	125.44	335.53	50.24	2189	2590	1673	5145	14882
RDF	2542	231.75	125.44	335.53	50.24	2189	2590	1673	5630	15367
Control	2542	231.75	125.44	335.53	50.24	2189	2590	1673	0	9737

Abbreviations: RDN: recommended dose of nitrogen, TD: top dressing; FS: foliar spray, NCU: neem coated urea, RDF: recommended dose of fertilizer, LCC: leaf colour chart, SY: seed yield

### Energy use indices

The energetics of different nitrogen management modules in mustard presented in the Table 6. The energy input under various N application methods did not exhibited much differences. Despite utilizing the more or less equal amount of total nitrogen, the plots received RDN_100_ with 2% foliar spray of urea or NCU showed highest energy output (95238 MJ ha^-1^) over their reduced levels of RDN_75_ with similar foliar spray treatments (U or NCU @ 2% and KNO_3_ @ 1.5%). The GS based N application recorded 7.25% greater energy output (93088 MJ ha^-1^) as compared to RDF whereas, LCC based N application was at par with RDF. The control treatment displayed lowest energy output among all the treatments. The net energy gain (energy output minus energy input) was higher under RDN_100_ + FS of NCU and urea @ 2% followed by GS based precise N application owing to their enhanced energy efficiency. The maximum energy use efficiency was accomplished by the administration of N through GS (6.51%) followed by RDN_75_ + FS as compared to RDN_100_ + FS. Further, RDF recorded lesser value of EUE (5.71%). Again, similar trend of pattern was followed with respect to energy profitability (Epfy) and the values were varied from 4.71 (lowest in RDF) to 5.51 (highest in GS based N application) under various N management modules. The values for direct energy (4927 MJ) and renewable energy consumption (2338 MJ) were same among all the treatments, whereas, these values were divergent under each treatment pertaining to indirect and non-renewable energy consumption. The direct energy consumption was highest under RDN_100_ than RDN_75_ along with FS of different N sources (U, NCU and KNO_3_) owing to the increased amount of nitrogen used, followed by GS sensor based N application. Similar trend of pattern was also followed with respect to non-renewable energy usage, whereas, control received least values of both these energies (IDE and NRE). We further observed that the highest energy productivity (18% higher) was achieved by the précised application of N through GreenSeeker (0.721 kg MJ^-1^) over RDF (0.611 kg MJ^-1^) and similarly RDN_75_ than RDN_100_ with foliar spray treatments. This indicates that the treatments with reduced application of N were resulted into the increased energy productivity. The specific energy was greater under RDF (1.64 MJ kg^-1^) followed by LCC based N and RDN_100_ + FS NCU @ 2% both equally as compared to other treatments whereas; lowest specific energy was registered under GS. Here, foliar spray of KNO_3_ @ 1.5% results better specific energy than FS of urea or NCU @ 2%. Correspondingly, the values of energy intensiveness were higher under RDN_100_ than RDN_75_ in conjunction with FS followed by RDF, GS and LCC. The unfertilized plot had lesser value of energy intensive (20.45 MJ USD^-1^). Congruently, human energy profitability was directly proportionate to the aggregate of N and higher with increased N levels than their reduced levels (RDN_100_ > RDN_75_) plus FS of different N sources (Urea, NCU and KNO_3_) and the augmentation was between 0.34 to 9.78%, respectively to the RDF. The treatments with lower levels of N have apprehensible conserved energy with reduced utilization over recommended practice (RDF). The percent variation in energy saving, in comparison to the recommended dose of fertilizer (RDF), ranged from +2.97% (modest energy saving) to −43.06% (significant energy losses that means required higher energy consumption) across different nitrogen application treatments (**Fig 4**). The highest energy saving (%) was registered under GS based N application (8.07%) after RDN_75_ + 1.5% KNO_3_ FS (10.13% due reduced N level) as compared to other N treatments, whereas control shows higher value of energy saving because of lack of fertilizer application. The foliar spray treatments required more energy than rest of the treatments.

**Table 6:** Influence of nitrogen management modules on energetics of *Brassica juncea* L. in two years experimentation (Pooled over two years).

Treatment	EI (MJ)	EO (MJ)	NE (MJ)	EUE (%)	Epfy	DE (MJ)	IDE (MJ)	RE (MJ)	NRE (MJ)	EP (kg MJ^-1^)	SE (MJ kg^-1^)	EI (MJ USD^-1^)	HEP
RDN_100_ (50% N basal + 50% N TD) + 2% Urea FS	15533	95238	79705	6.13	5.13	4927	10606	2338	13195	0.660	1.51	28.18	965
RDN_100_ (50% N basal + 50% N TD) + 2% NCU FS	15533	95588	80055	6.15	5.15	4927	10606	2338	13195	0.668	1.50	28.14	969
RDN_100_ (50% N basal + 50% N TD) + 1.5% KNO_3_ FS	15269	91975	76706	6.02	5.02	4927	10342	2338	12931	0.649	1.54	27.37	932
RDN_75_ (50% N basal + 25% N TD) + 2% Urea FS	14321	91450	77129	6.39	5.39	4927	9394	2338	11983	0.684	1.46	26.12	927
RDN_75_ (50% N basal + 25% N TD) + 2% NCU FS	14321	89613	75292	6.26	5.26	4927	9394	2338	11983	0.678	1.47	26.09	908
RDN_75_ (50% N basal + 25% N TD) + 1.5% KNO_3_ FS	14057	86063	72005	6.12	5.12	4927	9130	2338	11719	0.661	1.51	25.34	872
RDN – Green seeker	14289	93088	78799	6.51	5.51	4927	9362	2337	11952	0.721	1.39	26.56	943
RDN – LCC	14713	87075	72362	5.92	4.92	4927	9786	2337	12376	0.651	1.54	26.91	882
RDF	15198	86788	71590	5.71	4.71	4927	10271	2337	12861	0.611	1.64	27.73	879
Control	10350	66600	56250	6.43	5.43	4927	5423	2337	8013	0.679	1.47	20.45	675

Abbreviations: RDN: recommended dose of nitrogen, TD: top dressing; FS: foliar spray, NCU: neem coated urea, RDF: recommended dose of fertilizer, LCC: leaf colour chart, EI: Energy input; EO: Energy output; EUE: Energy use efficiency; NE: Net energy gain; EPfy: Energy profitability; DE: Direct energy; IDE: Indirect energy; RE: Renewable energy; NRE: Nonrenewable energy; EP: Energy productivity; SE: Specific energy; EI: Energy intensiveness; HEP: Human energy profitability

### Carbon footprint

The data on the C emission (Table 7) under various N management treatments revealed that majority of the portion was contributed by the fertilizers which range from 45.6 to 52.3%, respectivel (Fig 3)y, whereas other inputs has similar C emission values in total CO_2_ emissions across the treatments. The potential for global warming resulting from the use of various inputs in mustard seed production under a range of treatment combinations was emphasized. This potential was calculated by adding the total emissions of CO_2_ and N_2_O. In total CO_2_ emission, RDN_100_ with foliar spray of N recorded higher amount than RDN_75_. GS based N application has 8.07% lesser emission over RDF. Similarly, in case of N_2_O emission, GS based N application has least amount than rest of other N management treatments and 18.9% lesser N_2_O emission over recommended practices. Conversely, the yield-scaled carbon footprint (CFy) values varied from 111.2 (as minimum) to 139.0 kg CO_2_-eq. t^-1^ (as maximum) mustard biological yield among N management modules. However, the lowest value of the same was observed under control where no N application was made.

**Table 7:** Effect of nutrient management modules on carbon footprint of *Brassica juncea* L. under different agricultural operations (Pooled over two years).

Treatment	Land preparation & sowing	Seed	Diesel	Fertilizer	Plant protection chemicals	Labour	Machinery	Total CO_2_ (kg CO_2_-e ha^-1^)	N_2_O (kg CO_2_-e ha^-1^)	GWP (kg CO_2_-e ha^-1^)	CFy (kg CO_2_-e t^-1^)
RDN_100_ (50% N basal + 50% N TD) + 2% Urea FS	172.78	5.34	153	513	15.3	43	46.48	944	400	1344	131.0
RDN_100_ (50% N basal + 50% N TD) + 2% NCU FS	172.78	5.34	153	513	15.3	43	46.48	944	400	1344	129.5
RDN_100_ (50% N basal + 50% N TD) + 1.5% KNO_3_ FS	172.78	5.34	153	492	15.3	43	46.48	922	380	1302	131.3
RDN_75_ (50% N basal + 25% N TD) + 2% Urea FS	172.78	5.34	153	414	15.3	43	46.48	844	307	1151	117.5
RDN_75_ (50% N basal + 25% N TD) + 2% NCU FS	172.78	5.34	153	414	15.3	43	46.48	844	307	1151	118.5
RDN_75_ (50% N basal + 25% N TD) + 1.5% KNO_3_ FS	172.78	5.34	153	393	15.3	43	46.48	823	286	1109	119.4
RDN – Green seeker	172.78	5.34	153	412	15.3	43	46.48	842	304	1146	111.2
RDN – LCC	172.78	5.34	153	446	15.3	43	46.48	877	337	1214	126.6
RDF	172.78	5.34	153	486	15.3	43	46.48	916	375	1291	139.0
Control	172.78	5.34	153	89	15.3	43	46.48	519	0	519	74.0

Abbreviations: RDN: recommended dose of nitrogen, TD: top dressing; FS: foliar spray, NCU: neem coated urea, RDF: recommended dose of fertilizer, LCC: leaf colour, N_2_O: nitrogen oxide, CO_2_: carbon dioxide, GWP: global warming potential; chart, CFy: yield-scaled carbon footprint

A comparative analysis of carbon footprint across the nitrogen management modules were summarized in the Table 8. Results indicated that the highest spatial carbon footprint was ascribed by the treatments having N application at 100% of recommended level, i.e., RDN_100_ than RDN_75_ along with foliar spray of N @ 2% either through Urea or NCU followed by FS of KNO_3_ @ 1.5%. The extent of CO_2_ was approximately 81 per cent higher over unfertilized control treatment (1921 kg CO_2_-e ha^-1^) and about 3% greater over recommended practice (RDF) by following the former treatments. Whereas, the GS sensor based N application registered for minimum spatial carbon footprint (3101 kg CO_2_-e ha^-1^) among N application methods except treatment RDN_75_ (50% N basal + 25% N TD) + 1.5% KNO_3_ foliar spray with its reduced level of N. Similarly, higher values of carbon input, carbon output and Net C gain was observed with higher N levels. Application of N through GS recorded less C input (8.1% less), more C output (by 10.9%) and Net C gain (by 16.5%) over RDF, while, lesser values of these C indices were noted under unfertilized plot (control). Furthermore, the lowest carbon footprint (0.30 kg CE kg^-1^) was recorded under GS-based N application among the various N management treatments, whereas the highest value (0.39 kg CE kg^-1^) was observed under the RDF treatment. Additionally, the lesser carbon footprint results into higher carbon efficiency ratio (5.35) and carbon efficiency (3.37 kg kg^-1^ CE) as observed in case of GS based N management compared to RDF (4.43 & 2.54 kg kg^-1^ CE). Correspondingly, another important carbon index, known as Carbon Stability Index (CSI) was found to be higher under precise N application through GS, compared to a lower CSI value of 3.43 (less stable carbon dynamics) observed under the RDF treatment. This showed that GS-based precise N application not only reduced the carbon footprint but also enhanced carbon stability in the system. Moreover, the value of CSI was decreased with an increase of N levels across the treatments. The reduction in the carbon footprint value was observed highest (Fig 4) when N to the crop was applied using optical sensor based GS (5.98%) after RDN_75_ + 1.5% KNO_3_ FS (7.51%). However, the control plot recorded highest per cent reduction in CF (31.90%) due to absence of N application. This is evident that quantum of carbon footprint decreases with increasing levels of nitrogen. Similarly, the yield variations due to application of various N management options ranged between 2.73% (lowest with RDN_75_ + 1.5% KNO_3_ FS) to 22.02% (highest with GS) with maximum yield reduction (27.61%) in control plots (Fig 4c).

**Table 8:** Carbon dynamics measured through various carbon parameters in *Brassica juncea* L. as influenced by different nitrogen management modules (Pooled over two years).

Treatment	Spatial carbon footprints (kg CO_2_-e ha^-1^)	C input (kg CE ha^-1^)	C output (kg CE ha^-1^)	Net C gain (kg CE ha^-1^)	C footprint (kg CE kg^-1^ MY*)	C efficiency ratio	C efficiency (kg kg^-1^ CE)	CSI
RDN_100_ (50% N basal + 50% N TD) + 2% Urea FS	3473	949	4513	3564	0.36	4.76	2.78	3.76
RDN_100_ (50% N basal + 50% N TD) + 2% NCU FS	3473	949	4565	3616	0.35	4.81	2.88	3.81
RDN_100_ (50% N basal + 50% N TD) + 1.5% KNO_3_ FS	3394	927	4363	3436	0.36	4.70	2.76	3.70
RDN_75_ (50% N basal + 25% N TD) + 2% Urea FS	3110	850	4312	3462	0.34	5.07	2.92	4.07
RDN_75_ (50% N basal + 25% N TD) + 2% NCU FS	3110	850	4275	3425	0.33	5.03	3.00	4.03
RDN_75_ (50% N basal + 25% N TD) + 1.5% KNO_3_ FS	3031	828	4088	3260	0.34	4.94	2.91	3.94
RDN – Green seeker	3101	847	4535	3687	0.30	5.35	3.37	4.35
RDN – LCC	3228	882	4217	3335	0.34	4.78	2.97	3.78
RDF	3373	922	4086	3164	0.39	4.43	2.54	3.43
Control	1921	525	3091	2566	0.31	5.89	3.23	4.89

Abbreviations: RDN: recommended dose of nitrogen, TD: top dressing; FS: foliar spray, NCU: neem coated urea, RDF: recommended dose of fertilizer, LCC: leaf colour chart, CSI: carbon stability index, seed yield

## Discussion

### Yield attributes and yields

Synchronization between the nitrogen demand by the plant and supply from soil available N creates congenial environment to the plant growth and development which improve growth and yield characteristics of the crop. The enhancement in yield traits brought about by diverse N management tactic results in to more economic output and biomass production which eventually lead to higher monetary benefit. GS guided N application recorded maximum siliquae per plant (39.7 & 16.9% more), seed/sliquae (29.4 & 4.0% higher), 1000 seed weight (25.7 & 13.2% increase), respectively to the control. This could be because the crop was able to absorb more nutrients due to the increased availability of N in the soil and better synchronization may results into the reduced N losses occurs due to various means. The present findings were supported by the outcomes of earlier investigation [10,54,55]. In mustard, a general recommendation that has been proven to be successful in increasing seed yield and nutrient use efficiency is to apply N nitrogen in two splits, i.e., half as basal and half at first irrigation on 35−40 DAS. A substantial enhancement in mustard seed and stover yield was noticed which attributed to consistent N application made with the help of GreenSeeker crop sensor over rest of the treatments. The mean of two years yield data revealed 22.0 and 64.5% increase in mustard yield than recommended practice and control plot due to multiple N applications made in splits. A favourable environment to plant growth is established through improved soil fertility status brought about by increased soil nutrient availability and harmony between crop N necessity and soil supply through splitting application. Moreover, later developmental stages experience concurrent vegetative and reproductive portions due to enhanced photosynthetic partitioning [56]. When crop plants received adequate fertilization, their productivity increased because vital nutrients were continuously released and transferred to them [57]. Another advantage of applying nitrogen in many splits as it reduces the amount of N lost to the environment through different processes such as volatilization, leaching, denitrification, etc. Previous research indicated that these losses accounted for around 50% of the applied nitrogen. The crop eventually benefited from a decreased N loss because plants can absorb as much N as possible to enhance their growth and development. In the “Indo-Gangetic plains of South Asia,” the GS sensor based N application provided better yield in irrigated wheat by effectively regulating nitrogen, and it was determined that optical sensor-based N application could be the most important strategy for reaching greater NUE [58]. Similarly, two split application of nitrogen guided by GS^TM^ (GreenSeeker^TM^) improved maize grain yield by 22.4%, improved N energy use efficiency by 37.4%, reduced greenhouse gas emissions by 21.0%, and achieved a higher eco-efficiency index (0.19 US $ MJ^-1^ and 1.03 US $ kg^-1^) than SR. The carbon footprint on a yield scale (CFy) was reduced by 48.2%, indicating a substantial decrease in CO₂ emissions per economic yield unit. [59]. The basic idea behind this approach is to synchronize the nutritional requirement of the plant with nitrogen supply, therefore feeding the developing plants rather than the soil. Studies in the past also showed that applying lower amounts of N resulted in higher yields more quickly than applying higher quantities [60]. A positive correlation amid seed output and nitrogen splitting might be ascribed to elevated leaf N concentration, which promotes photosynthetic activity in plants, potentially contributing to augmented seed yield [14,61]. Due to the absence of fertilizer nutrients in the control treatment, it showed maximum percentage yield reduction compared to the general recommended practice (RDF).

### Monetary analysis

The economic analysis of *Brassica juncea* L. production under the investigated treatments was evaluated, and concluded that increasing input quantities together with other farm operations carried out according to conventional techniques, rises production expenses cumulatively whereas decreasing net profitability [62]. The enhancement in net returns was lower under control (US$ 561 ha^-1^) and higher under recommended practice (US$ 927 ha^-1^), suggesting that the cultivation of mustard was not sustainable economically or responsively. Due to more labour costs and fertilizer cumulative expenses, the cost of mustard production (US$ ha^-1^) under N foliar spray treatments was greater (by 2.02 to 3.70%) than under sensor guided N application. However, these values were reported lowest under control, where no fertilizer application was done. Administration of nitrogen using Greenseeker augmented the net profitability by 36.2% and 125.1% in comparison to the recommended practice and unfertilized treatment. Similar findings were also reported previous researchers [10,58]. Upadhyay et al. [59] also reported significant gain in the net monetary returns by 27.7 per cent and registered 28.9% more benefit cost ratio over state recommendations.

### Energy budgeting

A production system is deemed more efficient when, it utilizes lesser energy while producing a higher yield [63] in order to develop sustainable agriculture in a clean environment [64]. Irrespective of crops, earlier studies have also revealed that nutrient management in comparison to other agricultural inputs consumed more energy in the food production system [31,40,65] indicating major key role in crop husbandry to attain self-sufficiency in the food and feed. The analysis of energy inventory showed that the cultivation of *Brassica juncea* L. required a significant amount of energy, which was mostly contributed by nitrogen, diesel, threshers, and irrigation. The collective energy utilization from these inputs (nitrogen, diesel, thresher and irrigation) accounted for approximately half (46.2%) to the total energy requirement (Fig 2).

Earlier studies have also observed increased energy consumption in castor farming due to fuel, manure, and fertilizer use [66,67]. Plots applied with higher N levels, i.e., RDN_100_ along with FS of diverse N sources (Urea, NCU or KNO_3_) at 2% or 1.5% results in more energy input (15701 MJ ha^-1^) over other treatment plots. Across the treatments, the plots received N at recommended level (RDN_100_) associated with higher energy consumption than their reduced levels (N) at RDN_75_ owing to reduced levels of N application. Similarly, in foliar spray of N, the treatments with FS @ 1.5% through KNO_3_ attributed to lower energy consumption as compared to urea or NCU @ 2%. Since the energy coefficient of nitrogen is higher than that of phosphorus (11.11), potassium (6.70), zinc (20.90), and S (1.12) (Table 2), the amount of energy required to produce a crop increases significantly with each additional unit used. Even while RDF’s energy input was lower, its energy output (10%) was noticeably higher under N application with GS owing to an augmented biological yield. The increased energy efficiency can be ascribed to the superior energy production, which was due to the amplified net energy gain of aforesaid treatment, resulted in a considerable improvement in energy profitability. Conversely, the highest indirect energy utilization was noticed in treatments having RDN_100_ along with FS of N with urea or NCU @ 2% or KNO_3_ @ 1.5% followed by RDF, principally caused by the rising use of nitrogen fertilizers.. The consumption of renewable energy across treatments is more or less similar, whereas, non-renewable energy level varied across the treatments due to use of different nitrogen management methods. The use of a notably greater quantity of N in RDN_100_ with FS of N led to an increase in the utilization of non-renewable energy. The treatment with GS based N application indicated favourable energy profitability (5.51) in comparison to rest of the treatments because of greater energy gain. Earlier findings were also shown that different crops and farming techniques have better energy gains while maintaining energy profitability [31,40,65,66]. The combination of Nutrient Expert® and GreenSeeker™ warranted precise N application, reduces energy waste and optimizing resource use in maize [59].

### Carbon auditing

Today the concerns about life on Earth are being threatened by the intensifying levels of GHGs in the environment. In order to maintain the sustainability of agro-ecosystems, agro-techniques targeted at increasing agricultural output and profitability must also include carbon optimization tactics. In the present investigation, diverse nutrient management modules showed divergent carbon dynamics. It makes sense that higher levels of nutrient application would result in higher carbon use. In the study, results showed that application of nitrogen @ RDN_100_ along with FS of N through urea, NCU or KNO_3_ modules seems to be considerably carbon-costly N application tactic, registered 2.96% higher carbon input as compared to recommended practice (RDF). This might be due to combined effect of giving extra plant nutrition, and manpower usage. The augmented mustard yield required additional manpower for completing multi-harvests and post-harvest operations including processing, that eventually ascribed to elevated carbon input. Though, sensor based precise N application using GS reduced carbon input by 5.20% with an increase in carbon output and net C gain by 10.9% and 16.5%, respectively over RDF. Apart from this, other carbon indices like carbon footprint (23% less), carbon efficiency ratio (20.7% more), carbon efficiency (32.7% more) and carbon stability index (26.8%) were also improved through aforesaid N module due to application of reduced N rates (used only 65 kg/ha N instead of 80 kg/ha as in RDF). This may be because of greater carbon output ascribed to more biomass production of mustard. This indicates that these carbon indices were improved with decreasing levels of nitrogen application among the treatments. The crop has benefited greatly by being able to attain better economic yield and dry biomass production when crop N demand is synchronized with soil N supply and is available consistently through foliar feeding during crucial nutrient-demanding growth phases of the crop [59,65,66].

Overall, fertilizers and manures generally produced the highest CO_2_-e ha^-1^ emissions, followed by N_2_O emissions, which accounted for a substantial portion of the global warming potential. The third significant source contributing to GWP was the usage of diesel for various farm operations. When the magnitude of N grows and the C:N ratio rises, more greenhouse gases are released into the atmosphere [68–69]. In addition, a robust correlation was found between N_2_O emissions and applied nutrients. The comparable results were also observed by others [6,70]. This indicates that the carbon footprint is primarily associated with N dosages and has a significant impact on the crop’s ability to convert that nitrogen into yields [71]. The N management using GreenSeeker demonstrated about 21 per cent reduction in greenhouse gas emissions due to efficient nitrogen utilization over state recommendations [59]. These findings reinforce the importance of precision nutrient management in address ing productivity and environmental challenges in modern agriculture.

## Conclusion

Identifying carbon-efficient strategies through careful evaluation of emissions from various agricultural inputs can significantly reduce greenhouse gas (GHG) emissions associated with mustard cultivation. Beyond improving yield and profitability, the findings from this study offer valuable insights into sustainable nitrogen (N) management practices that support environmental safety, energy-efficient production systems, and GHG mitigation. The application of nitrogen using optical sensor based tool, i.e., GreenSeeker enhanced mustard productivity and improved profitability as compared to the RDF. This approach also achieved nitrogen saving of 18.7% by aligning nitrogen application with the crop’s real-time needs and synchronizing it with soil nitrogen supply. Among all agricultural inputs, four major contributors: fertilizer N, diesel, threshing, and irrigation accounted for nearly 46.25% of total energy use. The GS-based nitrogen application demonstrated improvements across several energy and carbon efficiency metrics when compared to RDF. It also resulted in a lower carbon footprint of 0.30 kg CE kg ⁻ ¹, compared to 0.39 kg CE kg ⁻ ¹ under RDF. Further, by reducing crop N requirement through precise N management in splits may also reduce the government expenditures on subsidies provided on N fertilizers. The results of this study may address the future research issues and guide policy decisions aimed at achieving climate-resilient and carbon-neutral agricultural systems. This study warrants that more field research on diverse nitrogen management strategies is needed to offer detailed insights into energy and carbon budgeting in mustard cultivation.
